# Editorial: Pathophysiology and pharmacological treatments associated with liver failures

**DOI:** 10.3389/fphar.2024.1407835

**Published:** 2024-04-12

**Authors:** Chandana B. Herath, Omar El Hiba, Xiang Zhou, Xuefeng Luo, Kaihui Lu

**Affiliations:** ^1^ Department of Medicine, University of Melbourne, Austin Health, Heidelberg, Australia; ^2^ Laboratory of Anthropogenic, Biotechnology and Health, Nutritional Physiopathologies, Neurosciences and Toxicology Team, Faculty of Sciences, Chouaib Doukkali University, El Jadida, Morocco; ^3^ Fudan University Shanghai Cancer Center and Institutes of Biomedical Sciences, Fudan University, Shanghai, China; ^4^ Department of Gastroenterology and Hepatology, West China Hospital, Sichuan University, Chengdu, China; ^5^ Division of Cardiology, Pulmonology and Vascular Medicine, Medical Faculty, Heinrich Heine University of Düsseldorf, Düsseldorf, Germany

**Keywords:** liver failures, Hepatology, liver cancer, hepatic encephalopathy, hepatic fibrosis, pathophysiology, pharmacology

This editorial summarizes the contributions to the Frontiers Research topic “pathophysiology and pharmacological treatments associated with liver failures” with peer-reviewed articles published in Frontiers in Pharmacology.

Liver diseases (LD), which refers to chronic and acute hepatic failures, represent one of the main global health threats on the human life with associated high mortality. Several etiologies have been identified with relevance to the onset of liver diseases and disease progression, including excess alcohol consumption, inflammatory bowel disease, obesity and metabolic disorders; particularly diabetes and insulin-resistance, autoimmune and viral (HBV, HCV) hepatitis, drugs such as acetaminophen overdose and some mycotoxins (eg. aflatoxins) (Forrest et al., 1979; Clark et al., 2002; Guidotti and Chisari, 2006; O’shea et al., 2010; Mekuria et al., 2023).

Based on these etiologies, liver diseases may be classified as viral hepatitis (HBV, HCV), non alcoholic fatty liver disease (NAFLD), alcoholic steatohepatitis (ASH), hemochromatosis, autoimmune liver disease, biliary atresia, Gilbert syndrome, liver cirrhosis and hepatocellular carcinoma. Epidemiological studies have demonstrated a wide variability of the prevalence of liver disease across countries depending on the lifestyle and other factors including hygiene (Teng et al., 2023). Indeed, liver diseases account for more than two million deaths annually, which constitutes approximately 4% of all deaths. Clinically, liver disease-associated deaths are largely attributable to complications of cirrhosis and/or cirrhosis progressing to hepatocellular carcinoma (Devarbhavi et al., 2023).

Whilst researchers have made a significant progress in developing techniques for disease diagnosis, improvement in the management of patients with liver disease still requires further research. Hence, the current Research Topic enabled hepatologists to highlight the latest developments in molecular and cellular pathogenesis of various liver diseases, as well as the relevant therapeutic strategies for the management of liver disease. The aim was therefore to bring together the latest findings on the pathogenesis of liver failure and to propose potential treatment options for acute or chronic liver failure. In that context, Li et al. described and classified drug-induced acute liver failure together with a summary of the underlying mechanisms of acetaminophen hepatotoxicity. The authors highlighted the current research and novel findings in the diagnosis, screening, and management of acetaminophen induced-liver injury with a discussion on the current challenges and potential future research on drug-induced acute liver failure. The Research Topic included an article by Yao et al. who have provided a comprehensive review to emphasize the significance of exosomes which are known to serve as messengers for transmitting information between tissues and as regulators in metabolism and hepatic fibrosis. The authors discussed the regulatory impact of exosomes in liver fibrosis and how they can be potentially modulated by natural plant-derived, endogenous and synthetic compounds in our search for novel therapies for the treatment of hepatic fibrosis.

It has been hypothesized that steroidal sapogenin is able to protect the liver. In this article collection, Rhogani et al. have reviewed the literature to show that a steroidal sapogenin, diosgenin, is able to protect the liver by inhibiting proinflammatory and oxidative pathways as well as by modulating gut microbiota. It further shows that diosgenin modulates lipid profile in conditions such as MAFLD which is often associated with liver injury. Moreover, the review also highlighted promising hepatoprotective role of diosgenin against several other conditions such as steatohepatitis, diabetes mellitus, liver fibrosis and hepatocellular carcinoma. Published reports suggest that testosterone is hepatoprotective. In this Research Topic, Amer et al. investigated the immune and metabolic treatment approach of testosterone in a mouse model of liver injury to provide experimental evidence that testosterone has hepatoprotective action in this model. In an acute and chronic models with liver fibrosis induced by carbon tetrachloride administration, the authors showed that testosterone reduced liver fibrosis which was associated with a reduction in serum IL-6 and liver IL-6 receptor levels. This was accompanied by increased INF-gamma, apparently secreted by natural killer cells, thereby suggesting an immune-modulatory effect of testosterone in liver fibrosis. Additionally, hepatoprotective potential of glycyrrhizin arginine (Gly-Arg) salt was investigated by Guo et al. who demonstrated that in cisplatin-induced acute liver injury and in primary murine hepatocytes, Gly-Arg salt reduced the protein levels of BECN1 and LC3-II/LC3-I. Moreover, cisplatin-induced formation of BECN1-xCT complex associated with inhibition of glutathione peroxidase-4 activity was reversed by Gly-Arg salt. Finally, Ilhan Ocak performed a 15-year retrospective study in 127 adult patients with acute liver failure receiving mono supportive extracorporeal therapy vs. dual supportive extracorporeal therapy. The study found that both therapies significantly improved renal and hepatic biochemical parameters, and blood ammonia level in patients with acute liver failure associated with grade III-IV hepatic encephalopathy. Compared to mono supportive extracorporeal therapy, dual supportive extracorporeal therapy was associated with improved hemodynamic stability, lactic acidosis and acid base balance. The study also showed that both therapies utilizing their own protocols have improved patients’ survival compared to previously published trials in patients with acute liver failure, and warrants further investigations to validate the protocols of supportive therapies developed by the authors.

The article collection in this Research Topic provides data obtained from both experimental and clinical studies along with comprehensive literature reviews of the pathophysiology in liver disease and liver failure. The studies published in these articles suggested several therapeutic strategies that may potentially be adapted for the treatment of liver diseases.
